# Supplementary material for: Characterization of Gut Microbiome in the Mud Snail Cipangopaludina cathayensis in Response to High-Temperature Stress
Source: Animals (Basel). 2022 Sep 9;12(18):2361. doi: 10.3390/ani12182361 (PMC9494996; doi:10.3390/ani12182361)
Supplement: Supplementary file 1 [file animals-12-02361-s001.zip › animals-1822408-supplementary.pdf]

Table S1. Number of sequences derived from 12 samples.

ID	Input	Filtered	Denoised	Merged	Nonchimeric	Nonsingleton
C1-1	88377	80648	79217	65048	61274	60944
C1-2	95129	83367	79397	72030	64258	63544
C1-3	90380	83698	83360	81033	80696	80675
T1-1	94555	81378	78043	71741	64516	63818
T1-2	188858	172564	167904	152696	138952	137854
T1-3	189346	171570	166971	159357	144429	143496
C2-1	90517	84329	83131	66931	61914	61669
C2-2	89798	84111	83187	81189	78551	78408
C2-3	96840	89648	89007	86148	84470	84405
T2-1	94250	81542	77829	69578	64464	64080
T2-2	93623	81120	78366	73521	67462	67118
T2-3	181050	158596	155311	147443	136045	135427

Table S2. Alpha-diversity indexes of different samples under a 97% similarity cut-off.

ID	Good's coverage	Shannon	Simpson
C1	0.997±0.004	6. 00±0.994	0.909±0.097
C2	0.999±0.000	7.135±0.148	0.964±0.005
T1	0.990±0.003	6.769±0.826	0.919±0.075
T2	0.994±0.002	5.465±0.606	0.818±0.101

Table S3. Results of PERMAVOVA of Bray-Curtis dissimilarity between samples using ANOSIM test.

Group1	Group2	Sample size	Permutations	R	*p*-value	*q*-value
all	-	12	999	0.635802	0.001	-
C1	C2	6	999	0.555556	0.098	0.1755
C1	T1	6	999	0.481481	0.202	0.214
C1	T2	6	999	0.333333	0.214	0.214
C2	T1	6	999	1	0.11	0.1755
C2	T2	6	999	1	0.117	0.1755
T1	T2	6	999	0.666667	0.098	0.1755

Table S4. Results of PERMAVOVA of Bray-Curtis dissimilarity between samples using Adonis test.

Index	Df	Sums Of Sqs	Mean Sqs	F. Model	R2	Pr(>F)
T	3	1.227025	0.409008	4.124162	0.607314	0.003
Residuals	8	0.793389	0.099174	NaN	0.392686	NaN
Total	11	2.020415	NaN	NaN	1	NaN

Table S5. Relative abundance of intestinal bacteria at the phylum level.

ID	C1	C2	T1	T2
Proteobacteria	65.04%	63.62%	46.26%	43.59%
Bacteroidetes	5.54%	8.73%	19.82%	3.64%
Firmicutes	3.34%	8.21%	6.94%	1.96%
Actinobacteria	2.44%	3.64%	1.42%	1.33%
Chlamydiae	0.55%	9.22%	0.36%	0.76%
Deinococcus-Thermus	0.64%	1.20%	1.30%	0.63%
Verrucomicrobia	0.44%	2.36%	0.23%	0.08%
Chloroflexi	1.67%	0.58%	0.52%	0.23%
Patescibacteria	0.59%	0.46%	0.13%	0.12%
Tenericutes	0.32%	0.06%	0.76%	0.06%
Other	19.43%	1.92%	22.26%	47.61%

Table S6. Relative abundance of intestinal bacteria at the genus level.

ID	C1	C2	T1	T2
Pseudomonas	13.43%	17.85%	11.95%	8.38%
Acinetobacter	8.74%	11.77%	5.54%	9.71%
Pelomonas	9.69%	3.44%	2.42%	6.73%
Bacteroides	0.21%	0.87%	14.65%	0.15%
Aeromonas	2.81%	4.79%	1.99%	0.78%
Aquabacterium	2.45%	1.93%	1.08%	2.88%
Rhodobacter	2.42%	2.91%	2.15%	0.85%
Halomonas	1.23%	0.00%	1.10%	2.81%
Muribaculaceae	0.41%	2.90%	0.82%	1.11%
Brevundimonas	1.25%	0.85%	0.48%	1.13%
Others	57.36%	52.69%	57.81%	65.47%

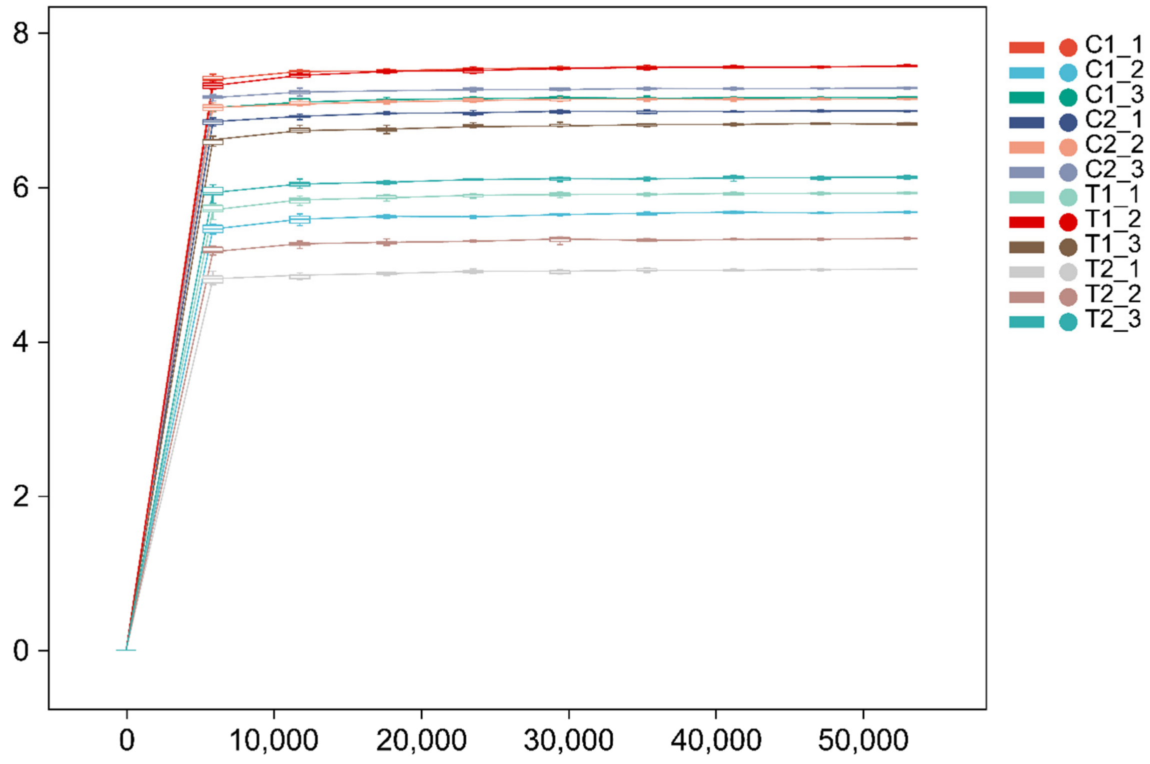

Figure S1. Rarefaction curves for all analyzed samples. Different samples are represented with different colors.

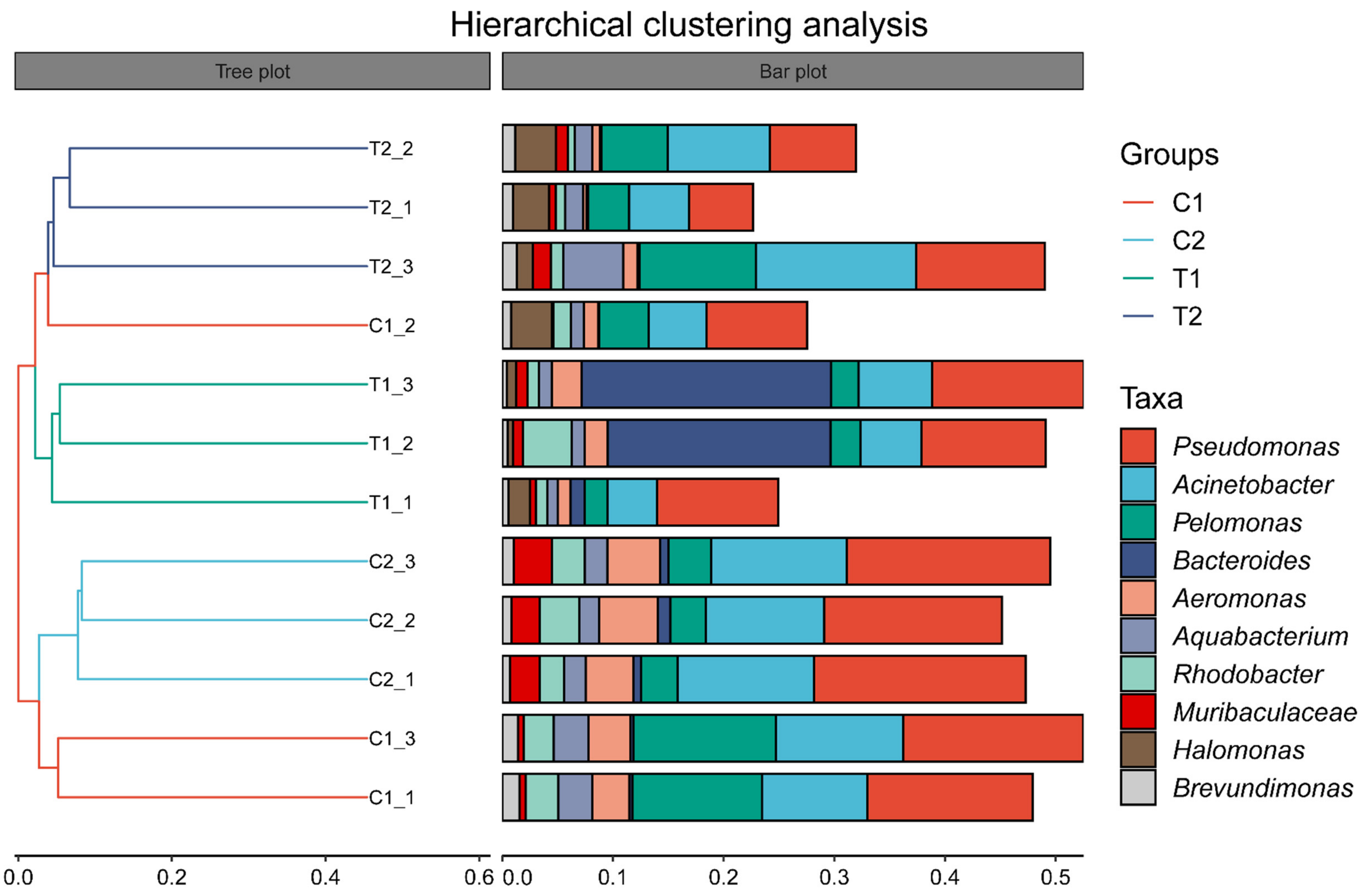

Figure S2. Hierarchical cluster analysis of the Jaccard distances generated from taxa tables showed Amplicon Sequence Variant (ASV) similarity across microbial communities among *Cipangopaludina cathayensis* treatment groups C1 (control treatment; 25 ± 1 °C, day 3), C2 (control treatment; 25 ± 1 °C, day 7), T1 (high-temperature treatment; 32 ± 1 °C, day 3), and T2 (high-temperature treatment; 32 ± 1 °C, day 7).
